# A century of trends in adult human height

**DOI:** 10.7554/eLife.13410

**Published:** 2016-07-26

**Authors:** NCD Risk Factor Collaboration (NCD-RisC), James Bentham

**Affiliations:** Imperial College London, United Kingdom; Middlesex University, United Kingdom; Imperial College London, United Kingdom; World Health Organization, Switzerland; Imperial College London, United Kingdom; Imperial College London, United Kingdom; World Health Organization, Switzerland; Imperial College London, United Kingdom; Imperial College London, United Kingdom; Harvard T.H. Chan School of Public Health, United States; Harvard T.H. Chan School of Public Health, United States; Harvard T.H. Chan School of Public Health, United States; World Health Organization, Switzerland; Indian Council of Medical Research, India; Imperial College London, United Kingdom; University of California, Berkeley, United States; Imperial College London, United Kingdom; Imperial College London, United Kingdom; WHO Collaborating Centre on NCD Surveillance and Epidemiology, United Kingdom; Al-Quds University, Palestine; Center for Diabetes and Endocrine Care, India; Birzeit University, Palestine; Instituto Mexicano del Seguro Social, Mexico; The University of Adelaide, Australia; Mahidol University, Thailand; Instituto Nacional de Ciencias Médicas y Nutricion, Mexico; University of Amsterdam, The Netherlands; Non-Communicable Diseases Research Center, Iran; Leibniz Institute for Prevention Research and Epidemiology - BIPS, Germany; King Saud University, Saudi Arabia; Kuwait Institute for Scientific Research, Kuwait; Ministry of Health, Saudi Arabia; World Health Organization Regional Office for the Eastern Mediterranean, Egypt; Luxembourg Institute of Health, Luxembourg; ISGlobal Centre for Research in Environmental Epidemiology, Spain; World Health Organization Regional Office for the Eastern Mediterranean, Egypt; Lille University and Hospital, France; London School of Hygiene & Tropical Medicine, United Kingdom; Sogn and Fjordane University College, Norway; Norwegian School of Sport Sciences, Norway; Madras Diabetes Research Foundation, India; National Institute of Public Health, Tunisia; Norwegian Institute of Public Health, Norway; Ministry of Health Malaysia, Malaysia; Indian Council of Medical Research, India; University of Strasbourg and Strasbourg University Hospital, France; University of Yaoundé 1, Cameroon; Regional Authority of Public Health, Banská Bystrica, Slovakia; Shahid Beheshti University of Medical Sciences, Iran; Indian Council of Medical Research, India; King Abdulaziz University, Saudi Arabia; Indian Council of Medical Research, India; Medical University of Gdansk, Poland; Universidad Autónoma de Madrid, Spain; University of Palermo, Italy; Pan American Health Organization, United States; Mohammed V University de Rabat, Morocco; University of Pernambuco, Brazil; Dalhousie University, Canada; Jordan University of Science and Technology, Jordan; Federal University of Maranhao, Brazil; University of Sydney, Australia; University of Auckland, New Zealand; University Tunis El Manar, Tunisia; University Medical Science, Cuba; Imperial College London, United Kingdom; Universidad Peruana Cayetano Heredia, Peru; Lithuanian University of Health Sciences, Lithuania; University of São Paulo, Brazil; B. J. Medical College, India; Chirayu Medical College, India; Sunder Lal Jain Hospital, India; The Aga Khan University, Pakistan; The Hospital for Sick Children, Canada; The Aga Khan University, Pakistan; Shandong University of Traditional Chinese Medicine, China; Shanghai Jiao-Tong University School of Medicine, China; University of Southern Denmark, Denmark; University of Greenland, Greenland; University of Oslo, Norway; University of Oslo, Norway; University of Gothenburg, Sweden; National Institute for Public Health and the Environment, The Netherlands; University of Turin, Italy; University College London, United Kingdom; Liverpool John Moores University, United Kingdom; Nanyang Technological University, Singapore; German Institute of Human Nutrition, Germany; CEMIC, Argentina; Toulouse University School of Medicine, France; Ministry of Health, Seychelles; University of Lausanne, Switzerland; Ghent University, Belgium; FrieslandCampina, Singapore; Universidad Central de Venezuela, Venezuela; World Health Organization, Switzerland; Bielefeld University, Germany; German Cancer Research Center, Germany; University of Amsterdam, The Netherlands; The Fred Hollows Foundation New Zealand, New Zealand; University of Turin, Italy; National Institute for Public Health and the Environment, The Netherlands; University of Southern Denmark, Denmark; Cork Institute of Technology, Ireland; Universidad de La Laguna, Spain; University of Malta, Malta; Ministry of Health, Tonga; Canadian Fitness and Lifestyle Research Institute, Canada; Hospital Santa Maria, CHLN; Istanbul University, Turkey; Universidade Federal de Juiz de Fora, Brazil; Cardiologia di Mercato S. Severino, Italy; University of São Paulo, Brazil; Karolinska Institutet, Sweden; University of Porto, Portugal; Santiago de Compostela University, Spain; University College London, United Kingdom; Associazione Calabrese di Epatologia, Italy; India Diabetes Research Foundation, India; Duke-NUS Graduate Medical School, Singapore; Imperial College London, United Kingdom; National Institute of Medical Statistics, India; University College London, United Kingdom; Academia Sinica, Taiwan; Capital Institute of Pediatrics, China; Duke University, United States; Kailuan General Hospital, China; University of Oxford, United Kingdom; Duke-NUS Graduate Medical School, Singapore; The Gertner Institute for Epidemiology and Health Policy Research, Israel; Lausanne University Hospital, Switzerland; Ministry of Health and Welfare, Taiwan; Victor Babes University of Medicine and Pharmacy Timisoara, Romania; Seoul National University College of Medicine, South Korea; Korea Centers for Disease Control and Prevention, South Korea; University of Southern Denmark, Denmark; Medical University of Silesia, Poland; Charles University in Prague, Czech Republic; Katholieke Universiteit Leuven, Belgium; Ghent University, Belgium; Agency for Preventive and Social Medicine, Austria; University of Southampton, United Kingdom; University College London, United Kingdom; Cork Institute of Technology, Ireland; IRCCS Istituto Neurologico Mediterraneo Neuromed, Italy; Institut Pasteur de Lille, France; Westmead University of Sydney, Australia; Canadian Fitness and Lifestyle Research Institute, Canada; CIBEROBN, Spain; National Council of Research, Italy; Federal University of Santa Catarina, Brazil; Institut Pasteur de Lille, France; Eduardo Mondlane University, Mozambique; University of Copenhagen, Denmark; Harvard TH Chan School of Public Health, United States; The Gertner Institute for Epidemiology and Health Policy Research, Israel; Lille University Hospital, France; Ghent University, Belgium; Ghent University, Belgium; IRCCS Istituto Neurologico Mediterraneo Neuromed, Italy; Ghent University, Belgium; Ghent University, Belgium; Madras Diabetes Research Foundation, India; National Research Centre for Preventive Medicine, Russia; Erasmus Medical Center Rotterdam, The Netherlands; University of Montreal, Canada; Institut de Recherche pour le Développement, France; French Public Health Agency, France; Erasmus Medical Center Rotterdam, The Netherlands; IRCCS Istituto Neurologico Mediterraneo Neuromed, Italy; Universidade do Vale do Rio dos Sinos, Brazil; National Council of Scientific and Technical Research, Argentina; Non-Communicable Diseases Research Center, Iran; National Institute of Nutrition, Vietnam; University of Queensland, Australia; Istituto Superiore di Sanità, Italy; Universidad de Cuenca, Ecuador; Helmholtz Zentrum München, Germany; Ministére de la Santé et de la Lutte Contre le Sida, Côte d’Ivoire; The Cardinal Wyszynski Institute of Cardiology, Poland; University of Latvia, Latvia, Europe; University of Benin, Nigeria; University of Gothenburg, Sweden; Norwegian School of Sport Sciences, Norway; National Institute of Nutrition and Food Technology, Tunisia; Imperial College London, United Kingdom; University of California Davis, United States; University of Stellenbosch, South Africa; Karadeniz Technical University, Turkey; University of Southern Denmark, Denmark; Instituto Mexicano del Seguro Social, Mexico; The Queen's University of Belfast, United Kingdom; University of Zurich, Switzerland; University of Southampton, United Kingdom; Tehran University of Medical Sciences, Iran; Centro de Salud Villanueva Norte, Spain; The University of the West Indies, Jamaica; Hospital Don Benito-Villanueva de la Serena, Spain; Ministry of Health, Argentina; Council for Agricultural Research and Economics, Italy; Pontificia Universidad Católica de Chile, Chile; Toulouse University School of Medicine, France; University of Manchester, United Kingdom; University of Tartu, Estonia; Instituto Nacional de Salud Pública, Mexico; Agency for Preventive and Social Medicine, Austria; Universiti Sains Malaysia, Malaysia; Luleå University, Sweden; Dalarna University, Sweden; Stanford University, United States; World Health Organization Regional Office for the Eastern Mediterranean, Egypt; The University of the West Indies, Jamaica; Federal University of São Paulo, Brazil; Erasmus Medical Center Rotterdam, The Netherlands; Hospital Universitario Son Espases, Spain; Hospital de Clinicas de Porto Alegre, Brazil; Universidade Federal do Rio Grande do Sul, Brazil; Kindai University Faculty of Medicine, Japan; Kyoto University, Japan; Medical University of Warsaw, Poland; Victor Babes University of Medicine and Pharmacy Timisoara, Romania; University of KwaZulu-Natal, South Africa; University of Sydney, Australia; Geneva University Hospitals, Switzerland; CIBER en Epidemiología y Salud Pública, Spain; Australian Bureau of Statistics, Australia; Wageningen University, The Netherlands; Non-Communicable Diseases Research Center, Iran; Istituto Superiore di Sanità, Italy; University of Insubria, Italy; Lille University Hospital, France; Lund University, Sweden; Nutrition Department, Ministry of Health, Israel; Federal University of Pelotas, Brazil; Universidad Politécnica de Madrid, Spain; The Andes Clinic of Cardio-Metabolic Studies, Venezuela; National Institute of Hygiene, Epidemiology and Microbiology, Cuba; Université de Lille 2, France; Norwegian Institute of Public Health, Norway; Institute for Clinical and Experimental Medicine, Czech Republic; Children's Memorial Health Institute, Poland; Alexander Technological Educational Institute, Greece; Dalhousie University, Canada; Jagiellonian University Medical College, Poland; University of Southern Denmark, Denmark; University of Turin, Italy; University of Novi Sad, Serbia; National Center of Cardiovascular Diseases, China; University of Ferrara, Italy; Singapore Eye Research Institute, Singapore; Icelandic Heart Association, Iceland; Universidad Icesi, Colombia; Geneva University Hospitals, Switzerland; State University of Montes Claros, Brazil; King's College London, United Kingdom; Icelandic Heart Association, Iceland; Imperial College London, United Kingdom; Capital Medical University, China; Capital Medical University, China; Healis - Sekhsaria Institute for Public Health, India; University of Ibadan, Nigeria; Children's Memorial Health Institute, Poland; Institute for Clinical Effectiveness and Health Policy, Argentina; University of Zurich, Switzerland; Danish Cancer Society Research Centre, Denmark; The University of the West Indies, Barbados; University College London, United Kingdom; Indian Council of Medical Research, India; Kyushu University, Japan; University of Sydney, Australia; Tulane University, United States; Academic Medical Center of University of Amsterdam, The Netherlands; National Institute of Public Health, Mexico; Oulu University Hospital, Finland; Chronic Diseases Research Center, Iran; Imperial College London, United Kingdom; University of Hong Kong, China; The Chinese University of Hong Kong, China; University of Western Australia, Australia; Erasmus Medical Center Rotterdam, The Netherlands; Fundación Oftalmológica de Santander, Colombia; Universidade Federal de Pelotas, Brazil; University of Oran 1, Algeria; The University of the West Indies, Barbados; Independent Public Health Specialist, Myanmar; University of Oslo, Norway; International Realtions Division, Nay Pyi Taw; Peking University Health Science Center, China; Birzeit University, Palestine; National Institute of Nutrition, Vietnam; International Agency for Research on Cancer, France; American University of Beirut, Lebanon; IRCCS Istituto Neurologico Mediterraneo Neuromed, Italy; Cardiologia di Mercato S. Severino, Italy; Cairo University, Egypt; National Institute of Health and Nutrition, Japan; Erasmus Medical Center Rotterdam, The Netherlands; Institute for Clinical Effectiveness and Health Policy, Argentina; Aga Khan University, Pakistan; UHC Zagreb, Croatia; Niigata University, Japan; University of Auckland, New Zealand; Hadassah University Medical Center, Israel; Duke-NUS Graduate Medical School, Singapore; Kuwait Institute for Scientific Research, Kuwait; University of Adelaide, Australia; Norwegian University of Science and Technology, Norway; Jagiellonian University Medical College, Poland; University of Zagreb School of Medicine, Croatia; Guangzhou 12th Hospital, China; Simon Fraser University, Canada; International Agency for Research on Cancer, France; Ruprecht-Karls-University of Heidelberg, Germany; Research Centre for Prevention and Health, Denmark; World Health Organization Country Office, India; National Institute for Health and Welfare, Finland; University of Ljubljana, Slovenia; University of Zagreb, Croatia; German Cancer Research Center, Germany; University of Crete, Greece; The Gertner Institute for Epidemiology and Health Policy Research, Israel; Hellenic Medical Association for Obesity, Greece; Tehran University of Medical Science, Iran; Johns Hopkins Bloomberg School of Public Health, United States; National Institute of Epidemiology, India; Erasmus Medical Center Rotterdam, The Netherlands; University of Münster, Germany; Israel Center for Disease Control, Israel; Oulu University Hospital, Finland; Research Institute for Primordial Prevention of Non Communicable Disease, Iran; VU University Medical Center, The Netherlands; South African Medical Research Council, South Africa; Research Institute of Child Nutrition, Germany; University of Oxford, United Kingdom; Jordan University of Science and Technology, Jordan; Shahid Beheshti University of Medical Sciences, Iran; Seoul National University, South Korea; University of Cambridge, United Kingdom; FrieslandCampina, Singapore; Medical University Innsbruck, Austria; Muhimbili University of Health and Allied Sciences, Tanzania; National Cancer Center, South Korea; Statistics Austria, Austria; Lithuanian University of Health Sciences, Lithuania; B P Koirala Institute of Health Sciences, Nepal; Norwegian School of Sport Sciences, Norway; Institute of Tropical Medicine, Belgium; Tartu University Clinics, Estonia; National Institute for Health and Welfare, Finland; Kindai University Faculty of Medicine, Japan; Polish Academy of Sciences Anthropology Unit in Wroclaw, Poland; University Hospital Ulm, Germany; Norwegian University of Science and Technology, Norway; Wageningen University, The Netherlands; North-West University, South Africa; National Institute of Public Health, Czech Republic; University of Jyväskylä, Finland; Medical University of Lodz, Poland; The Children's Memorial Health Institute, Poland; Amrita Institute of Medical Sciences, India; The Cardinal Wyszynski Institute of Cardiology, Poland; All India Institute of Medical Sciences, India; National Institute for Health and Welfare, Finland; African Population and Health Research Center, Kenya; Higher Institute of Nursing Professions and Technical Health, Morocco; National Institute for Health and Welfare, Finland; Ghent University, Belgium; National Institute of Public Health of Algeria, Algeria; University of Hong Kong, China; Ministerio de Salud Pública, Cuba; Institute for Clinical and Experimental Medicine, Czech Republic; Sahlgrenska Academy, Sweden; Endocrinology and Metabolism Research Center, Iran; Norwegian University of Science and Technology, Norway; Indian Council of Medical Research, India; National Institute of Nutrition, Vietnam; National Institute of Nutrition, Vietnam; Food and Agriculture Organization, Italy; National University of Singapore, Singapore; National Cancer Center, South Korea; Tampere University Hospital, Finland; Universiti Putra Malaysia, Malaysia; Universidad Autónoma de Madrid, Spain; Harvard TH Chan School of Public Health, United States; West Virginia University, United States; National University of Singapore, Singapore; Oswaldo Cruz Foundation Rene Rachou Research Institute, Brazil; National Taiwan University, Taiwan; University of Chinese Academy of Sciences, China; Research Centre for Prevention and Health, Denmark; University of Gothenburg, Sweden; The Children's Memorial Health Institute, Poland; Beijing Anzhen Hospital, Capital Medical University; University Medicine Greifswald, Germany; University of São Paulo, Brazil; Consejería de Sanidad Junta de Castilla y León, Spain; Lithuanian University of Health Sciences, Lithuania; National Institute for Health and Welfare, Finland; Universidade do Porto, Portugal; University of Uppsala, Sweden; Peking University, China; Peking University, China; Universidade Federal de Ouro Preto, Brazil; The Jikei University School of Medicine, Japan; National Research Council, Italy; Baker IDI Heart and Diabetes Institute, Australia; Institut de Recherche pour le Développement, France; Hospital Israelita Albert Einstein, Brazil; Tehran University of Medical Sciences, Iran; Duke-NUS Graduate Medical School, Singapore; Indian Council of Medical Research, India; Institute of Internal and Preventive Medicine, Russia; Harokopio University, Greece; University of Otago, New Zealand; University of Padova, Italy; Pontificia Universidad Católica de Chile, Chile; University of Reading, United Kingdom; Lausanne University Hospital, Switzerland; Institut Hospital del Mar d'Investigacions Médiques, Spain; Institut de Recherche pour le Développement, France; Emory University, United States; Sher-i-Kashmir Institute of Medical Sciences, India; UiT The Arctic University of Norway, Norway; Cape Peninsula University of Technology, South Africa; University of Rzeszow, Poland; University of Yaoundé 1, Cameroon; The University of the West Indies, Jamaica; Brown University, United States; London School of Hygiene & Tropical Medicine, United Kingdom; University of Edinburgh, United Kingdom; University of Otago, New Zealand; University College Dublin, Ireland; Universiti Teknologi MARA, Malaysia; University of Oran 1, Algeria; Institut National de la Santé et de la Recherche Médicale, France; Helmholtz Zentrum München, Germany; Universidade Federal de Pelotas, Brazil; Robert Koch Institute, Germany; Indian Council of Medical Research, India; University of Tartu, Estonia; Capital Institute of Pediatrics, China; University of Copenhagen, Denmark; University of Tartu, Estonia; University of Otago, New Zealand; Pontificia Universidad Católica de Chile, Chile; Universidad Peruana Cayetano Heredia, Peru; University of Zagreb, Croatia; Ain Shams University, Egypt; Tehran University of Medical Sciences, Iran; Isfahan Cardiovascular Research Center, Iran; Madras Diabetes Research Foundation, India; Ministry of Health Malaysia, Malaysia; University of Copenhagen, Denmark; University of Southern Denmark, Denmark; University of Pécs, Hungary; Mulago Hospital, Uganda; Instituto Nacional de Salud Pública, Mexico; University of Limpopo, South Africa; Universidade Federal do Rio Grande do Sul, Brazil; University Medical Science, Cuba; Universidad de Zaragoza, Spain; RCSI Dublin, Ireland; University of Copenhagen, Denmark; Harokopio University, Greece; International Institute of Molecular and Cell Biology, Poland; Ain Shams University, Egypt; University of Porto, Portugal; Ahvaz Jundishapur University of Medical Sciences, Iran; Gorgas Memorial Institute of Public Health, Panama; Department of Public Health, Myanmar; University of Brescia, Italy; Helmholtz Zentrum München, Germany; Imperial College London, United Kingdom; University of Eastern Finland, Finland; Mary Immaculate College, Ireland; Universiti Sains Malaysia, Kota Bharu; University of Zagreb, Croatia; Ulm University, Germany; Kobe University, Japan; Regional Authority of Public Health, Banska Bystrica; National University of Singapore, Singapore; Suraj Eye Institute, India; Helen Keller International, Cameroon; Healis - Sekhsaria Institute for Public Health, India; CIBER en Epidemiología y Salud Pública, Spain; West Virginia University, United States; Jagiellonian University Medical College, Poland; Karolinska Institutet, Sweden; Pontificia Universidad Católica de Chile, Chile; Robert Koch Institute, Germany; University of Pharmacy and Medicine of Ho Chi Minh City, Vietnam; Hanoi Medical University, Vietnam; Universidad Centro-Occidental Lisandro Alvarado, Venezuela; Shanghai Jiao-Tong University School of Medicine, China; Kyushu University, Japan; Heartfile, Pakistan; National Research Council, Italy; Imperial College London, United Kingdom; University of Palermo, Italy; Eastern Mediterranean Public Health Network, Jordan; The Queen's University of Belfast, United Kingdom; Korea Centers for Disease Control and Prevention, South Korea; Kuwait Institute for Scientific Research, Kuwait; University of Vale do Rio dos Sinos, Brazil; National Food and Nutrition Institute, Poland; Ministry of Health Malaysia, Malaysia; Istanbul University, Turkey; Pan American Health Organization, United States; University of Puerto Rico, Puerto Rico; Research Center for Prevention and Health, Denmark; MRC Lifecourse Epidemiology Unit, United Kingdom; University of Novi Sad, Serbia; Fundación Oftalmológica de Santander, Colombia; Aarhus University, Denmark; Kwame Nkrumah University of Science and Technology, Ghana; Institute for Social and Preventive Medicine, Switzerland; University of Coimbra, Portugal; University of Latvia, Latvia; Jagiellonian University Medical College, Poland; Cancer Prevention and Research Institute, Italy; University of Wisconsin-Madison, United States; Istituto Superiore di Sanità, Italy; Ruprecht-Karls-University of Heidelberg, Germany; University of Bari, Italy; University of Otago, New Zealand; Tehran University of Medical Sciences, Iran; University of Zagreb, Croatia; Healis - Sekhsaria Institute for Public Health, India; University Medical Center Utrecht, The Netherlands; Oswaldo Cruz Foundation Rene Rachou Research Institute, Brazil; National Institute for Health and Welfare, Finland; Heart Institute, Brazil; University of Puerto Rico, Puerto Rico; Helmholtz Zentrum München, Germany; Lithuanian University of Health Sciences, Lithuania; Non-Communicable Diseases Research Center, Iran; Vietnam National Heart Institute, Vietnam; Leibniz Institute for Prevention Research and Epidemiology - BIPS, Germany; University College London, United Kingdom; Federal Ministry of Health, Bosnia and Herzegovina; Cardiovascular Prevention Centre, Italy; University Hospital of Pisa, Italy; University of New South Wales, Australia; The Cardinal Wyszynski Institute of Cardiology, Poland; Public Health Agency of Catalonia, Spain; Universiti Kebangsaan Malaysia, Malaysia; Institut Hospital del Mar d'Investigacions Médiques, Spain; Erasmus Medical Center Rotterdam, The Netherlands; Alexander Technological Educational Institute, Greece; Madras Diabetes Research Foundation, India; Indian Council of Medical Research, India; University of Edinburgh, United Kingdom; Victor Babes University of Medicine and Pharmacy Timisoara, Romania; University Clinics, Estonia; Al-Quds University, Palestine; Alborz University of Medical Sciences, Iran; Ministry of Health, Vietnam; University of Novi Sad, Serbia; Lithuanian University of Health Sciences, Lithuania; Institute of Epidemiology Disease Control and Research, Bangladesh; Turku University Hospital, Finland; Amrita Institute of Medical Sciences, India; National Institute of Epidemiology, India; India Diabetes Research Foundation, India; University of New South Wales, Australia; Institut Universitari d’Investigació en Atenció Primària Jordi Gol, Spain; University of Malaya, Malaysia; Karolinska Institutet, Sweden; University of Valencia, Spain; University of the Philippines, Philippines; Minas Gerais State Secretariat for Health, Brazil; Imperial College London, United Kingdom; Health Center San Agustín, Spain; PharmAccess Foundation, The Netherlands; Hospital Israelita Albert Einstein, Brazil; Instituto Nacional de Salud Pública, Mexico; University of Southampton, United Kingdom; Public Health Agency of Canada, Canada; Universidad Autónoma de Madrid, Spain; Canarian Health Service, Spain; Universidad Industrial de Santander, Colombia; Instituto Nacional de Salud Pública, Mexico; Mahidol University, Thailand; CIBEROBN, Spain; University of Eastern Finland, Finland; University of Gothenburg, Sweden; Fiji National University, Fiji; Institute for Clinical Effectiveness and Health Policy, Argentina; University of Zurich, Switzerland; University of Madeira, Portugal; Instituto Mexicano del Seguro Social, Mexico; Heart Institute, Brazil; Medical University of Gdansk, Poland; Singapore Eye Research Institute, Singapore; Sitaram Bhartia Institute of Science and Research, India; Faculty of medicine of Tunis, Tunisia; French Public Health Agency, France; National Institute of Public Health, Mexico; National Institute for Health and Welfare, Finland; University of Helsinki, Finland; University of Brescia, Italy; National Institute of Health, Peru; Ministry of Health, Indonesia; Catalan Department of Health, Spain; Universidade de Lisboa, Portugal; Institute of Preventive Medicine and Public Health, Portugal; University of Sao Paulo Clinics Hospital, Brazil; University of Porto, Portugal; South Karelia Social and Health Care District, Finland; Universidade de Lisboa, Portugal; Isfahan Cardiovascular Research Center, Iran; German Cancer Research Center, Germany; Research and Education Institute of Child Health, Cyprus; University of Sao Paulo Clinics Hospital, Brazil; Robert Koch Institute, Germany; Hospital Italiano de Buenos Aires, Argentina; Robert Koch Institute, Germany; Rigshospitalet, Denmark; Federal University of Santa Catarina, Brazil; Academic Medical Center of University of Amsterdam, The Netherlands; MRC North-West University, South Africa; Ministry of Health, Myanmar; Norwegian University of Science and Technology, Norway; Lagos State University College of Medicine, Nigeria; Digestive Diseases Research Institute, Iran; National Research Centre for Preventive Medicine, Russia; B P Koirala Institute of Health Sciences, Nepal; Baker IDI Heart and Diabetes Institute, Australia; The University of Tokyo, Japan; Seoul National University College of Medicine, South Korea; Singapore Eye Research Institute, Singapore; Finnish Institute of Occupational Health, Finland; Singapore Eye Research Institute, Singapore; American University of Beirut, Lebanon; Federal University of Maranhao, Brazil; Federal University of Santa Catarina, Brazil; India Diabetes Research Foundation, India; St Vincent's Hospital, Australia; University of New South Wales, Australia; Karolinska Institutet, Sweden; Medical University of Lodz, Poland; International Institute of Molecular and Cell Biology, Poland; London School of Hygiene & Tropical Medicine, United Kingdom; University of Oxford, United Kingdom; Academic Medical Center of University of Amsterdam, The Netherlands; The Chinese University of Hong Kong, China; University of Yaoundé 1, Cameroon; Umeå University, Sweden; Health Polytechnics Institute, Indonesia; University of Bari, Italy; Lund University, Sweden; Peking University, China; University of Copenhagen, Denmark; University of Zagreb, Croatia; Institut Régional de Santé Publique, West Africa; University of Bordeaux, France; University of Leuven, Belgium; University of Ljubljana, Slovenia; INSERM, France; University of Zurich, Switzerland; Heart Foundation, Australia; Norwegian School of Sport Sciences, Norway; Bonn University, Germany; Emory University, United States; Sotiria Hospital, Greece; Hadassah University Medical Center, Israel; Helmholtz Zentrum München, Germany; Helmholtz Zentrum München, Germany; Lund University, Sweden; National Institute of Public Health-National Institute of Hygiene, Poland; Swansea University, United Kingdom; University of Amsterdam, The Netherlands; Federal University of São Paulo, Brazil; Fu Jen Catholic University, Taiwan; Uppsala University, Sweden; The Chinese University of Hong Kong, China; ISGlobal Centre for Research in Environmental Epidemiology, Spain; Mahidol University, Thailand; The University of Auckland, New Zealand; University of the Philippines, Philippines; National Food and Nutrition Institute, Poland; National University of Singapore, Singapore; University of Tartu, Estonia; Lithuanian University of Health Sciences, Lithuania; Research Centre for Prevention and Health, Denmark; Peking University Health Science Center, China; University of KwaZulu-Natal, South Africa; Peking University, China; Ministry of Health, Jordan; University of Southern Denmark, Denmark; National Institute of Health, Peru; The University of Adelaide, Australia; UNICEF, Cameroon; Karolinska Institutet, Sweden; University of Leuven, Belgium; Research Centre for Prevention and Health, Denmark; Danish Cancer Society Research Centre, Denmark; National Institute for Health and Welfare, Finland; University of Southern Denmark, Denmark; Karadeniz Technical University, Turkey; Jagiellonian University Medical College, Poland; IB-SALUT Area de Salut de Menorca, Spain; University of Bologna, Italy; Institut de Recherche pour le Développement, France; Hellenic Health Foundation, Greece; Harvard TH Chan School of Public Health, United States; University of Pharmacy and Medicine of Ho Chi Minh City, Vietnam; Government Medical College, India; Sefako Makgatho Health Science University, South Africa; The University of the West Indies, Jamaica; University of Eastern Finland, Finland; Dasman Diabetes Institute, Kuwait; Ministry of Health, New Zealand; Karolinska Institutet, Sweden; Hellenic Medical Association for Obesity, Greece; University of Bordeaux, France; Harvard TH Chan School of Public Health, United States; Meharry Medical College, United States; Medical University of Innsbruck, Austria; Dokuz Eylul University, Turkey; University of Tampere Tays Eye Center, Finland; Pontificia Universidad Católica de Chile, Chile; University of Porto, Portugal; Harvard TH Chan School of Public Health, United States; University Medical Center Utrecht, The Netherlands; Ghent University, Belgium; Hanoi School of Public Health, Vietnam; University Medical Center Utrecht, The Netherlands; Academic Medical Center of University of Amsterdam, The Netherlands; Katholieke Universiteit Leuven, Belgium; Centro di Prevenzione Cardiovascolare Udine, Italy; Norwegian University of Science and Technology, Norway; Consejería de Sanidad Junta de Castilla y León, Spain; Universidade Federal de Minas Gerais, Brazil; University of Insubria, Italy; National Institute for Public Health and the Environment, The Netherlands; Institute of Tropical Medicine, Belgium; Universidade Federal de Pelotas, Brazil; Italian National Research Council, Italy; National Institute for Public Health and the Environment, The Netherlands; Finnish Institute of Occupational Health, Finland; Imperial College London, United Kingdom; Universidad Miguel Hernandez, Spain; University of Eastern Finland, Finland; INSERM, France; Ministry of Health, Seychelles; Lausanne University Hospital, Switzerland; University of Eastern Finland, Finland; UHC Zagreb, Croatia; ISGlobal Centre for Research in Environmental Epidemiology, Spain; University of the Witwatersrand, South Africa; University of Strasbourg, France; University College Cork, Ireland; Institute for Medical Research, Malaysia; Public Health Agency of Canada, Canada; Xinjiang Medical University, China; Beijing Tongren Hospital, China; University College London, United Kingdom; University of Cambridge, United Kingdom; Ministry of Health, New Zealand; St George’s, University of London; Medical University of Vienna, Austria; Universitas Indonesia, Indonesia; Medical University of Silesia, Poland; National Institute for Public Health and the Environment, The Netherlands; The University of the West Indies, Jamaica; Medical University Innsbruck, Austria; UiT The Arctic University of Norway, Norway; National Institute of Public Health-National Institute of Hygiene, Poland; Universiti Kebangsaan Malaysia, Malaysia; Duke-NUS Graduate Medical School, Singapore; The Chinese University of Hong Kong, China; University of Sydney, Australia; University of Oxford, United Kingdom; University of Manchester, United Kingdom; Shandong University of Traditional Chinese Medicine, China; Kailuan General Hospital, China; Institute of Food and Nutrition Development of Ministry of Agriculture, China; Capital Medical University, China; Mahidol University, Thailand; Children's Hospital of Fudan University, China; Chinese Center for Disease Control and Prevention, China; Ministry of Health, Turkey; University of Chinese Academy of Sciences, China; University of Cyprus, Cyprus; Niigata University, Japan; Capital Medical University, China; The University of the West Indies, Jamaica; Ministry of Health Malaysia, Malaysia; Universiti Teknologi MARA, Malaysia; University of Padova, Italy; Medical University of Gdansk, Poland; Duke University, United States; Beijing Anzhen Hospital, Capital Medical University; Chinese Center for Disease Control and Prevention, China; Singapore Eye Research Institute, Singapore; Chinese Center for Disease Control and Prevention, China; Inner Mongolia Medical University, China; Bispebjerg and Frederiksberg Hospitals, Denmark; Gorgas Memorial Institute of Public Health, Panama; McGill University, Canada

**Keywords:** biological sciences, medical research, epidemiology, nutrition, None

## Abstract

Being taller is associated with enhanced longevity, and higher education and earnings. We reanalysed 1472 population-based studies, with measurement of height on more than 18.6 million participants to estimate mean height for people born between 1896 and 1996 in 200 countries. The largest gain in adult height over the past century has occurred in South Korean women and Iranian men, who became 20.2 cm (95% credible interval 17.5–22.7) and 16.5 cm (13.3–19.7) taller, respectively. In contrast, there was little change in adult height in some sub-Saharan African countries and in South Asia over the century of analysis. The tallest people over these 100 years are men born in the Netherlands in the last quarter of 20th century, whose average heights surpassed 182.5 cm, and the shortest were women born in Guatemala in 1896 (140.3 cm; 135.8–144.8). The height differential between the tallest and shortest populations was 19-20 cm a century ago, and has remained the same for women and increased for men a century later despite substantial changes in the ranking of countries.

**DOI:** http://dx.doi.org/10.7554/eLife.13410.001

## eLife digest

People from different countries grow to different heights. This may be partly due to genetics, but most differences in height between countries have other causes. For example, children and adolescents who are malnourished, or who suffer from serious diseases, will generally be shorter as adults. This is important because taller people generally live longer, are less likely to suffer from heart disease and stroke, and taller women and their children are less likely to have complications during and after birth. Taller people may also earn more and be more successful at school. However, they are also more likely to develop some cancers.

The NCD Risk Factor Collaboration set out to find out how tall people are, on average, in every country in the world at the moment, and how this has changed over the past 100 years. The analysis revealed large differences in height between countries. The tallest men were born in the last part of the 20th century in the Netherlands, and were nearly 183 cm tall on average. The shortest women were born in 1896 in Guatemala, and were on average 140 cm tall. The difference between the shortest and tallest countries is about 20 cm for both men and women. This means there are large differences between countries in terms of nutrition and the risk of developing some diseases.

The way in which height has changed over the past 100 years also varies from country to country. Iranian men born in 1996 were around 17 cm taller than those born in 1896, and South Korean women were 20 cm taller. In other parts of the world, particularly in South Asia and parts of Africa, people are only slightly taller than 100 years ago, and in some countries people are shorter than they were 50 years ago.

There is a need to better understand why height has changed in different countries by different amounts, and use this information to improve nutrition and health across the world. It would also be valuable to understand how much becoming taller has been responsible for improved health and longevity throughout the world.

**DOI:** http://dx.doi.org/10.7554/eLife.13410.002

## Introduction

Being taller is associated with enhanced longevity, lower risk of adverse pregnancy outcomes and cardiovascular and respiratory diseases, and higher risk of some cancers (Paajanen et al., 2010; Emerging Risk Factors Collaboration, 2012; Green et al., 2011; Nelson et al., 2015; Batty et al., 2010; World Cancer Research Fund / American Institute for Cancer Research, 2007; 2010; 2011; 2012; 2014a; 2014b; Nüesch et al., 2015; Davies et al., 2015; Zhang et al., 2015; Kozuki et al., 2015; Black et al., 2008). There is also evidence that taller people on average have higher education, earnings, and possibly even social position (Adair et al., 2013; Stulp et al., 2015; Barker et al., 2005; Strauss and Thomas, 1998; Chen and Zhou, 2007; Case and Paxson, 2008).

Although height is one of the most heritable human traits (Fisher, 1919; Lettre, 2011), cross-population differences are believed to be related to non-genetic, environmental factors. Of these, foetal growth (itself related to maternal size, nutrition and environmental exposures), and nutrition and infections during childhood and adolescence are particularly important determinants of height during adulthood (Cole, 2000; Silventoinen et al., 2000; Dubois et al., 2012; Haeffner et al., 2002; Sørensen et al., 1999; Victora et al., 2008; Eveleth and Tanner, 1990; Tanner, 1962; Tanner, 1992; Bogin, 2013). Information on height, and its trends, can therefore help understand the health impacts of childhood and adolescent nutrition and environment, and of their social, economic, and political determinants, on both non-communicable diseases (NCDs) and on neonatal health and survival in the next generation (Cole, 2000; Tanner, 1992; Tanner, 1987).

Trends in men’s height have been analysed in Europe, the USA, and Japan for up to 250 years, using data on conscripts, voluntary military personnel, convicts, or slaves (Cole, 2000; Floud et al., 1990; Fogel et al., 1983; Schmidt et al., 1995; Floud et al., 2011; Tanner et al., 1982; Hatton and Bray, 2010; Tanner, 1981; Facchini and Gualdi-Russo, 1982). There are fewer historical data for women, and for other regions where focus has largely been on children and where adult data tend to be reported at one point in time or over short periods (Subramanian et al., 2011; Grasgruber et al., 2014; Baten and Blum, 2012; Deaton, 2007; Mamidi et al., 2011; van Zanden et al., 2014). In this paper, we pooled worldwide population-based data to estimate height in adulthood for men and women born over a whole century throughout the world.

## Results

**Figure 1.**
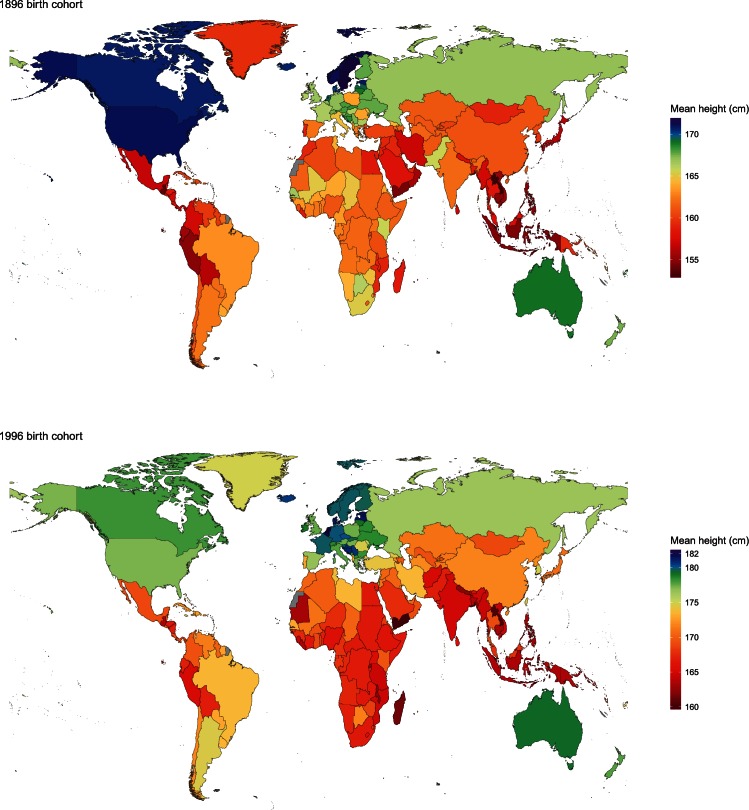
Adult height for the 1896 and 1996 birth cohorts for men. See www.ncdrisc.org for interactive version. **DOI:** http://dx.doi.org/10.7554/eLife.13410.003

**Figure 2.**
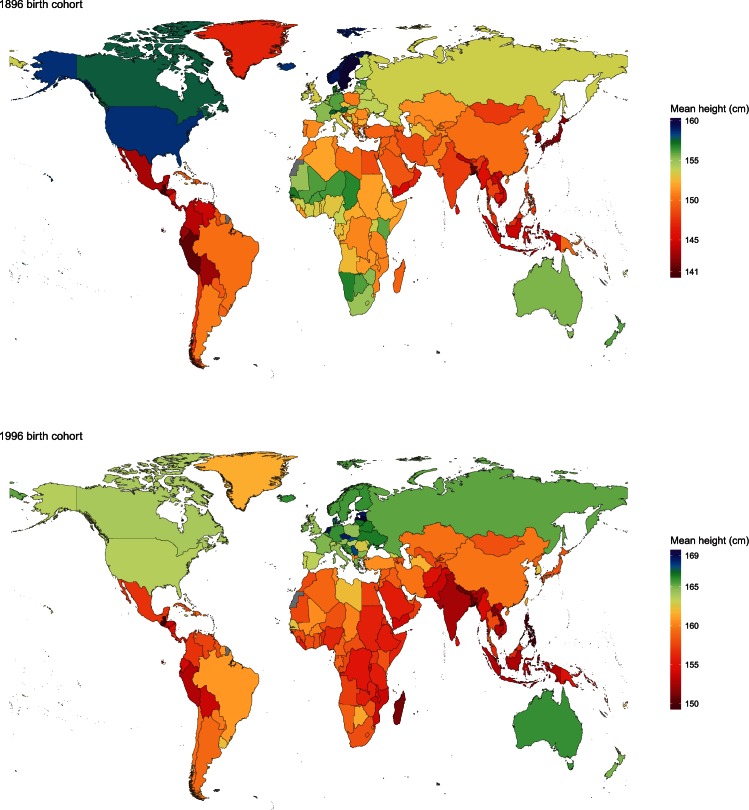
Adult height for the 1896 and 1996 birth cohorts for women. See www.ncdrisc.org for interactive version. **DOI:** http://dx.doi.org/10.7554/eLife.13410.004

We estimated that people born in 1896 were shortest in Asia and in Central and Andean Latin America (Figure 1 and Figure 2). The 1896 male birth cohort on average measured only 152.9 cm (credible interval 147.9–157.9) in Laos, which is the same as a well-nourished 12.5-year boy according to international growth standards (de Onis et al., 2007), followed by Timor-Leste and Guatemala. Women born in the same year in Guatemala were on average 140.3 cm (135.8–144.8), the same as a well-nourished 10-year girl. El Salvador, Peru, Bangladesh, South Korea and Japan had the next shortest women. The tallest populations a century ago lived in Central and Northern Europe, North America and some Pacific islands. The height of men born in Sweden, Norway and the USA surpassed 171 cm, ~18–19 cm taller than men in Laos. Swedish women, with average adult height of 160.3 cm (158.2–162.4), were the tallest a century ago and 20 cm taller than women in Guatemala. Women were also taller than 158 cm in Norway, Iceland, the USA and American Samoa.

**Figure 3.**
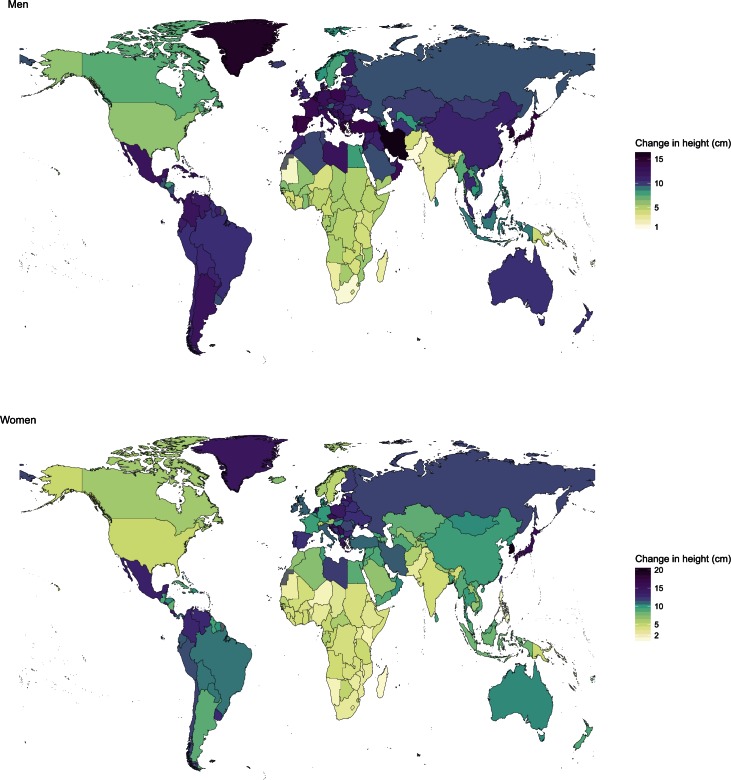
Change in adult height between the 1896 and 1996 birth cohorts. **DOI:** http://dx.doi.org/10.7554/eLife.13410.005

**Figure 4.**
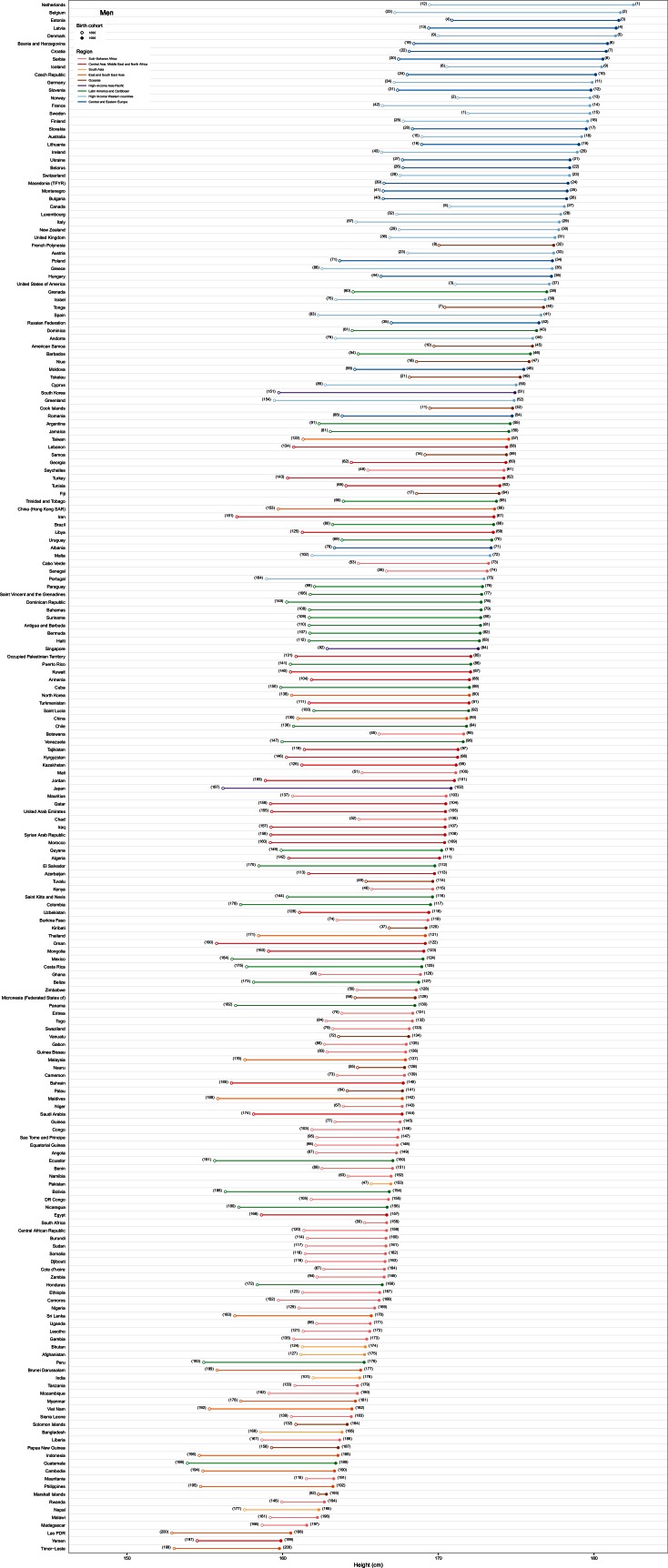
Height in adulthood for the 1896 and 1996 birth cohorts for men. The open circle shows the adult height attained by the 1896 birth cohort and the filled circle that of the 1996 birth cohort; the length of the connecting line represents the change in height over the century of analysis. The numbers next to each circle show the country’s rank in terms of adult height for the corresponding cohort. See www.ncdrisc.org for interactive version. **DOI:** http://dx.doi.org/10.7554/eLife.13410.006

**Figure 5.**
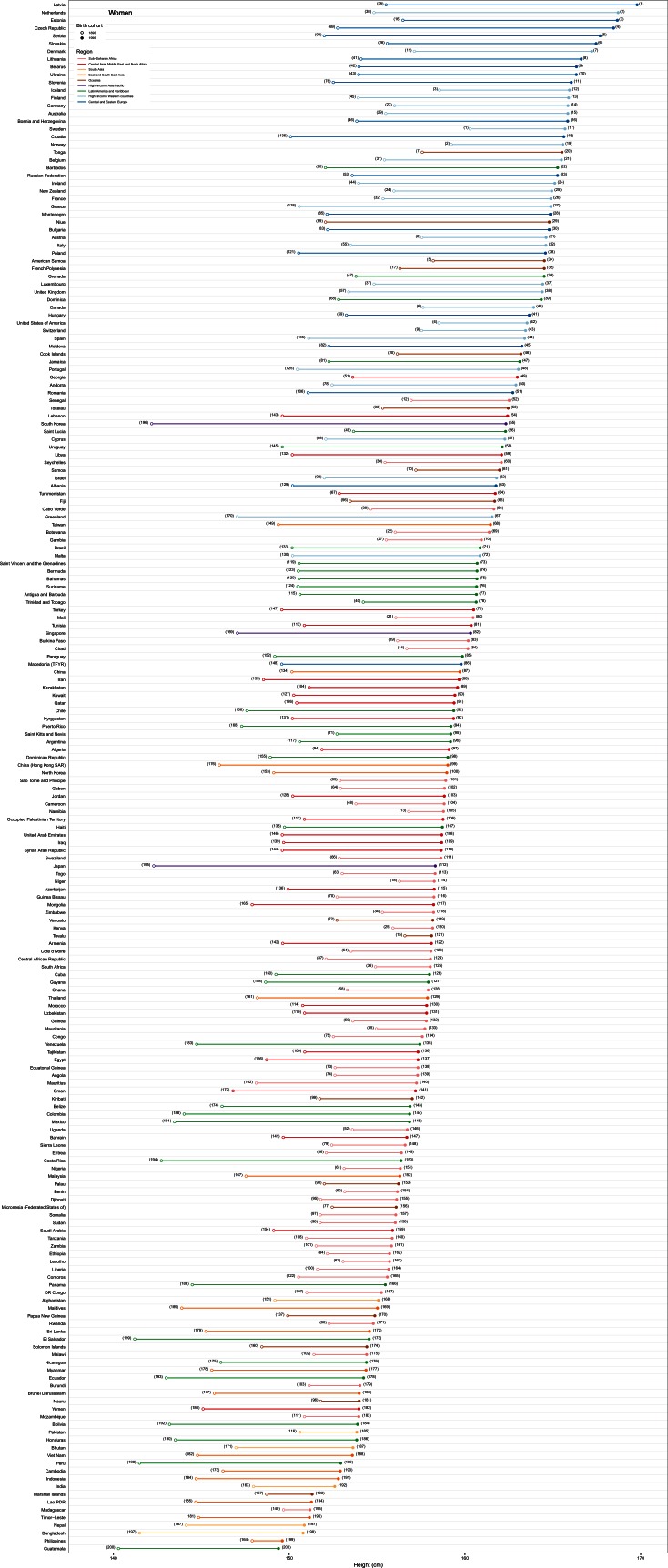
Height in adulthood for the 1896 and 1996 birth cohorts for women. The open circle shows the adult height attained by the 1896 birth cohort and the filled circle that of the 1996 birth cohort; the length of the connecting line represents the change in height over the century of analysis. The numbers next to each circle show the country’s rank in terms of adult height for the corresponding cohort. See www.ncdrisc.org for interactive version. **DOI:** http://dx.doi.org/10.7554/eLife.13410.007

Changes in adult height over the century of analysis varied drastically across countries. Notably, although the large increases in European men’s heights in the 19th and 20th century have been highlighted, we found that the largest gains since the 1896 birth cohort occurred in South Korean women and Iranian men, who became 20.2 cm (17.5–22.7) and 16.5 cm (13.3–19.7) taller, respectively (Figure 3, Figure 4 and Figure 5). As a result, South Korean women moved from the fifth shortest to the top tertile of tallest women in the world over the course of a century. Men in South Korea also had large gains relative to other countries, by 15.2 cm (12.3–18.1). There were also large gains in height in Japan, Greenland, some countries in Southern Europe (e.g., Greece) and Central Europe (e.g., Serbia and Poland, and for women Czech Republic). In contrast, there was little gain in height in many countries in sub-Saharan Africa and South Asia.

**Figure 6.**
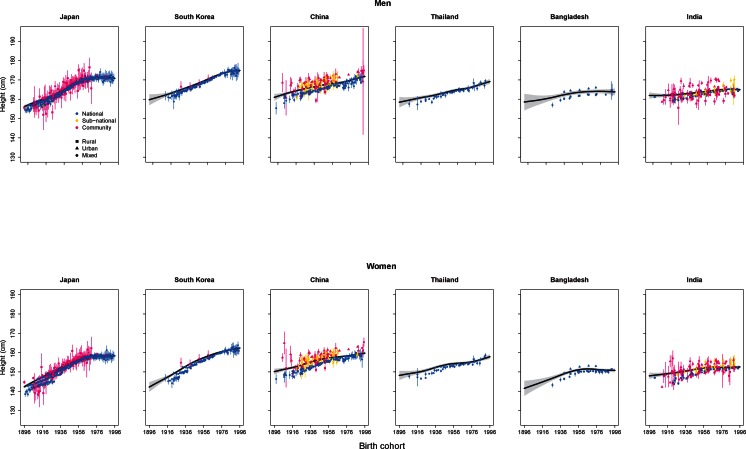
Trends in height for the adult populations of selected countries in Asia. The solid line represents the posterior mean and the shaded area the 95% credible interval of the estimates. The points show the actual data from each country, together with its 95% confidence interval due to sampling. The solid line and shaded area show estimated height at 18 years of age, while the data points show height at the actual age of measurement. The divergence between estimates and data for earlier birth cohorts is because participants from these birth cohorts were generally older when their heights were measured. **DOI:** http://dx.doi.org/10.7554/eLife.13410.008

The pace of growth in height has not been uniform over the past century. The impressive rise in height in Japan stopped in people born after the early 1960s (Figure 6). In South Korea, the flattening began in the cohorts born in the 1980s for men and it may have just begun in women. As a result, South Korean men and women are now taller than their Japanese counterparts. The rise is continuing in other East and Southeast Asian countries like China and Thailand, with Chinese men and women having surpassed the Japanese (but not yet as tall as South Koreans). The rise in adult height also seems to have plateaued in South Asian countries like Bangladesh and India at much lower levels than in East Asia, e.g., 5–10 cm shorter than it did in Japan and South Korea.

**Figure 7.**
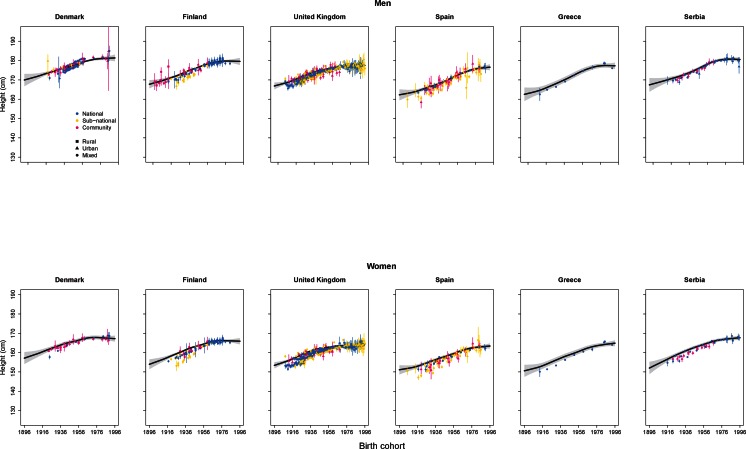
Trends in height for the adult populations of selected countries in Europe. The solid line represents the posterior mean and the shaded area the 95% credible interval of the estimates. The points show the actual data from each country, together with its 95% confidence interval due to sampling. The solid line and shaded area show estimated height at 18 years of age, while the data points show height at the actual age of measurement. The divergence between estimates and data for earlier birth cohorts is because participants from these birth cohorts were generally older when their heights were measured. **DOI:** http://dx.doi.org/10.7554/eLife.13410.009

There were also variations in the time course of height change across high-income western countries, with height increase having plateaued in Northern European countries like Finland and in English-speaking countries like the UK for 2–3 decades (Larnkaer et al., 2006; Schönbeck et al., 2013), followed by Eastern Europe (Figure 7). The earliest of these occurred in the USA, which was one of the tallest nations a century ago but has now fallen behind its European counterparts after having had the smallest gain in height of any high-income country (Tanner, 1981; Komlos and Lauderdale, 2007; Komlos and Baur, 2004; Sokoloff and Villaflor, 1982). In contrast, height is still increasing in some Southern European countries (e.g., Spain), and in many countries in Latin America.

**Figure 8.**
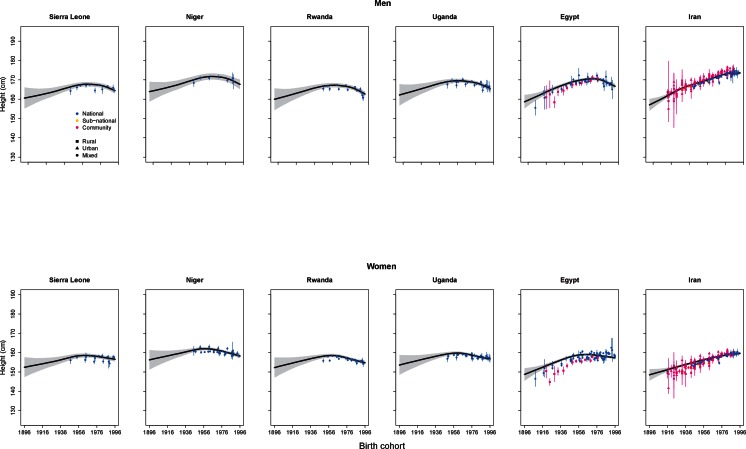
Trends in height for the adult populations of selected countries in the Middle East, North Africa, and sub-Saharan Africa. The solid line represents the posterior mean and the shaded area the 95% credible interval of the estimates. The points show the actual data from each country, together with its 95% confidence interval due to sampling. The solid line and shaded area show estimated height at 18 years of age, while the data points show height at the actual age of measurement. The divergence between estimates and data for earlier birth cohorts is because participants from these birth cohorts were generally older when their heights were measured. **DOI:** http://dx.doi.org/10.7554/eLife.13410.010

As an exception to the steady gains in most countries, adult height decreased or at best remained the same in many countries in sub-Saharan Africa for cohorts born after the early 1960s, by around 5 cm from its peak in some countries (see for example Niger, Rwanda, Sierra Leone, and Uganda in Figure 8). More recently, the same seems to have happened for men, but not women, in some countries in Central Asia (e.g., Azerbaijan and Uzbekistan) and Middle East and North Africa (e.g., Egypt and Yemen), whereas in others (e.g., Iran) both sexes continue to grow taller.

Men born in 1996 surpass average heights of 181 cm in the Netherlands, Belgium, Estonia, Latvia and Denmark, with Dutch men, at 182.5 cm (180.6–184.5), the tallest people on the planet. The gap with the shortest countries – Timor-Leste, Yemen and Laos, where men are only ~160 cm tall – is 22–23 cm, an increase of ~4 cm on the global gap in the 1896 birth cohort. Australia was the only non-European country where men born in 1996 were among the 25 tallest in the world. Women born in 1996 are shortest in Guatemala, with an average height of 149.4 cm (148.0–150.8), and are shorter than 151 cm in the Philippines, Bangladesh and Nepal. The tallest women live in Latvia, the Netherlands, Estonia and Czech Republic, with average height surpassing 168 cm, creating a 20 cm global gap in women’s height (Figure 5).

**Figure 9.**
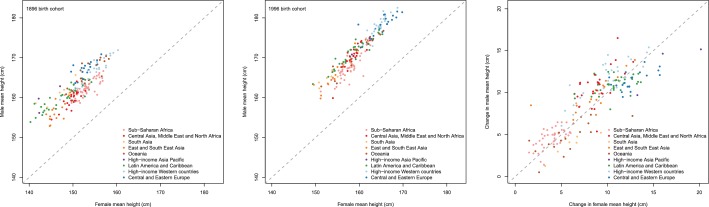
Height in adulthood for men vs. women for the 1896 and 1996 birth cohorts, and change in men’s vs. women’s heights from 1896 to 1996. **DOI:** http://dx.doi.org/10.7554/eLife.13410.011

Male and female heights were correlated across countries in 1896 as well as in 1996. Men were taller than women in every country, on average by ~11 cm in the 1896 birth cohort and ~12 cm in the 1996 birth cohort (Figure 9). In the 1896 birth cohort, the male-female height gap in countries where average height was low was slightly larger than in taller nations. In other words, at the turn of the 20th century, men seem to have had a relative advantage over women in undernourished compared to better-nourished populations. A century later, the male-female height gap is about the same throughout the height range. Changes in male and female heights over the century of analysis were also correlated, which is in contrast to low correlation between changes in male and female BMIs as reported elsewhere (NCD Risk Factor Collaboration, 2016).

**Figure 10.**
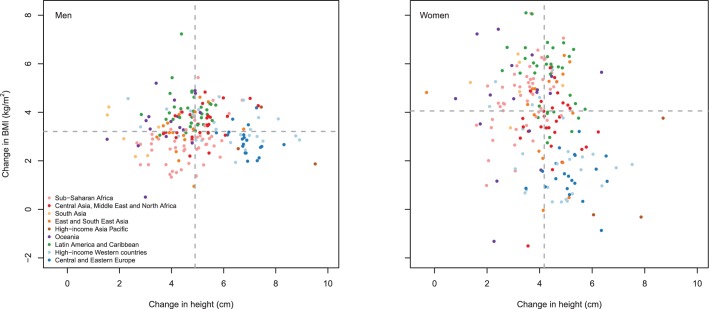
Change, over the 1928–1967 birth cohorts, in mean BMI vs. in mean height. Each point shows one country. BMI change was calculated for mean BMI at 45–49 years of age – an age when diseases associated with excess weight become common but weight loss due to pre-existing disease is still uncommon. BMI data were available for 1975–2014 (NCD Risk Factor Collaboration, 2016); 45–49 year olds in these years correspond to 1928–1967 birth cohorts. BMI data were from a pooled analysis of 1698 population-based measurement studies with 19.2 million participants, with details reported elsewhere (NCD Risk Factor Collaboration, 2016). **DOI:** http://dx.doi.org/10.7554/eLife.13410.012

Change in population mean height was not correlated with change in mean BMI (NCD Risk Factor Collaboration, 2016) across countries for men (correlation coefficient = −0.016) and was weakly inversely correlated for women (correlation coefficient = −0.28) (Figure 10). Countries like Japan, Singapore and France had larger-than-median gains in height but little change in BMI, in contrast to places like the USA and Kiribati where height has increased less than the worldwide median while BMI has increased a great deal.

## Discussion

We found that over the past century adult height has changed substantially and unevenly in the world’s countries, with no indication of convergence across countries. The height differential between the tallest and shortest populations was ~19 cm for men and ~20 cm for women a century ago, and has remained about the same for women and increased for men a century later despite substantial changes in the ranking of countries in terms of adult height.

Data from military conscripts and personnel have allowed reconstructing long-term trends in height in some European countries and the USA, albeit largely for men, and treating it as a 'mirror' to social and environmental conditions that affect nutrition, health and economic prosperity, in each generation and across generations (Tanner, 1987; Fogel, 2004; Komlos, 2009; Martins et al., 2014; Martorell, 1995). Our results on the large gains in continental European countries, and that they have overtaken English-speaking countries like the USA, are consistent with these earlier studies although these earlier analyses covered fewer countries in Eastern and Southern Europe, and used some self-reported data with simple adjustments that cannot fully correct for their bias (Hatton and Bray, 2010; Facchini and Gualdi-Russo, 1982; Baten and Blum, 2012).

Less has been known about trends in women’s height, and those in non-English-speaking/non-European parts of the world. We found that some of the most important changes in height have happened in these under-investigated populations. In particular, South Korean and Japanese men and women, and Iranian men, have had larger gains than European men, and similar trends are now happening in China and Thailand. These gains may partially account for the fact that women in Japan and South Korea have achieved the first and fourth highest life expectancy in the world (see also below). In contrast to East Asia’s impressive gains, the rise in height seems to have stopped early in South Asia and reversed in Africa, reversing or diminishing Africa’s earlier advantage over Asia. Prior studies have documented a rise in stunting in children in sub-Saharan Africa which continued to the mid-1990s (Stevens et al., 2012). Our results indicate that such childhood adversity may have carried forward to adulthood and be affecting health in the region. The early African advantage over Asia may also have been partly due to having a more diverse diet compared to the vegetable and cereal diet in Asia, partly facilitated by lower population density (Deaton, 2007; Moradi, 2010). Rising population, coupled with worsening economic status during structural adjustment, may have undermined earlier dietary advantage (Stevens et al., 2012; Pongou et al., 2006; Weil et al., 1990; Sundberg, 2009).

The main strengths of our study are its novel scope of estimating a century of trends in adult height for all countries in the world and for both sexes. Our population-based results complement the individual-level studies on the genetic and environmental determinants of within-population variation in height, and will help develop and test hypotheses about the determinants of adult height, and its health consequences. We achieved this by using a large number of population-based data sources from all regions of the world. We put particular emphasis on data quality and used only population-based data that had measured height, which avoids bias in self-reported height. Data were analysed according to a common protocol before being pooled, and characteristics and quality of data sources were verified through repeated checks by Collaborating Group members. Finally, we pooled data using a statistical model that could characterize non-linear trends and that used all available data while giving more weight to national data than to subnational and community surveys.

Although we have gathered an unprecedentedly comprehensive database of human height and growth, and have applied a statistical model that maximally utilizes the information in these sources, data in some countries were rather limited or were from community or sub-national studies. This is reflected in larger uncertainty of the estimated height in these countries. To overcome this, surveillance of growth, which has focused largely on children, should also systematically monitor adolescents and adults given the increasingly abundant evidence on their effects on adult health and human capital. Even measured height data can be subject to measurement error depending on how closely study protocols are followed. Finally, we did not have separate data on leg and trunk lengths, which may differ in their determinants, especially in relation to age at menarche and pre- vs. post-pubertal growth and nutrition, and health effects (Tanner et al., 1982; Frisch and Revelle, 1971).

**Figure 11.**
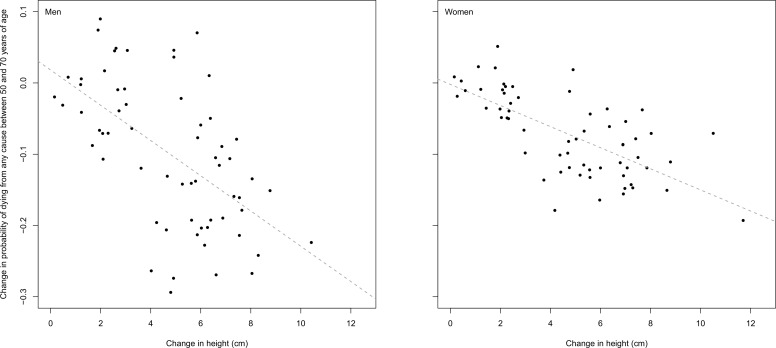
Association between change in probability of dying from any cause between 50 and 70 years of age and change in adult height by country for cohorts born between 1898 and 1946. Probability of death was calculated using a cohort life table. Mortality data were available for 1950 to 2013. The 1898 birth cohort is the first cohort whose mortality experience at 50–54 years of age was seen in the data, and the 1946 birth cohort the last cohort whose mortality experience at 65–69 years of age was seen in the data. The dotted line shows the linear association. The 62 countries included have vital registration that is >80% complete and have data on all-cause mortality for at least 30 cohorts. The countries are Argentina, Australia, Austria, Azerbaijan, Belarus, Belgium, Belize, Brazil, Bulgaria, Canada, Chile, China (Hong Kong SAR), Colombia, Costa Rica, Croatia, Cuba, Czech Republic, Denmark, Estonia, Finland, France, Germany, Greece, Guatemala, Hungary, Iceland, Ireland, Israel, Italy, Japan, Kazakhstan, Kyrgyzstan, Latvia, Lithuania, Luxembourg, Macedonia (TFYR), Malta, Mauritius, Mexico, Moldova, Netherlands, New Zealand, Norway, Poland, Portugal, Puerto Rico, Romania, Russian Federation, Slovakia, Slovenia, South Korea, Spain, Sweden, Switzerland, Trinidad and Tobago, Turkmenistan, Ukraine, United Kingdom, United States of America, Uruguay, Uzbekistan and Venezuela. **DOI:** http://dx.doi.org/10.7554/eLife.13410.013

Greater height in adulthood is both beneficially (cardiovascular and respiratory diseases) and harmfully (colorectal, postmenopausal breast and ovarian cancers, and possibly pancreatic, prostate and premenopausal breast cancers) associated with several diseases, independently of its inverse correlation with BMI (Paajanen et al., 2010; Emerging Risk Factors Collaboration, 2012; Green et al., 2011; Nelson et al., 2015; Batty et al., 2010; World Cancer Research Fund / American Institute for Cancer Research, 2007; 2010; 2011; 2012; 2014a; 2014b; Nüesch et al., 2015; Davies et al., 2015; Zhang et al., 2015). If the associations in epidemiological studies are causal, which is supported by the more recent evidence from Mendelian randomisation studies (Green et al., 2011; Nüesch et al., 2015; Davies et al., 2015; Zhang et al., 2015), the ~20 cm height range in the world is associated with a 17% lower risk of cardiovascular mortality and 20–40% higher risk of various site-specific cancers, in tall versus short countries. Consistent with individual-level evidence on the association between taller height and lower all-cause mortality in adult ages (Emerging Risk Factors Collaboration, 2012), gains in mean population height in successive cohorts are associated with lower mortality in middle and older ages in countries with reliable mortality data (correlation coefficient = −0.58 for men and −0.68 for women) (Figure 11), demonstrating the large impacts of height gain on population health and longevity. Further, short maternal stature increases the risk of small-for-gestational-age and preterm births, both risk factors for neonatal mortality, and of pregnancy complications (Kozuki et al., 2015; Black et al., 2008). Therefore, improvements vs. stagnation in women’s height can influence trends in infant and maternal mortality.

Our study also shows the potential for using height in early adulthood as an indicator that integrates across different dimensions of sustainable human development. Adult height signifies not only foetal and early childhood nutrition, which was included in the Millennium Development Goals, but also that of adolescents (Lancet, 2014). Further, adult height is a link between these early-life experiences and NCDs, longevity, education and earnings. It can easily be measured in health surveys and can be used to investigate differences across countries and trends over time, as done in our work, as well as within-country inequalities. Therefore, height in early adulthood, which varies substantially across countries and over time, provides a measurable indicator for sustainable development, with links to health and longevity, nutrition, education and economic productivity.

## Materials and methods

### Overview

We estimated trends in mean height for adults born from 1896 to 1996 (i.e., people who had reached their 18th birthday from 1914 to 2014) in 200 countries and territories. Countries were organized into 20 regions, mostly based on a combination of geography and national income (Supplementary file 1). Our study had two steps, described below. First, we identified, accessed, and re-analysed population-based measurement studies of human anthropometry. We then used a statistical model to estimate trends for all countries and territories.

### Data sources

We used data sources that were representative of a national, subnational, or community population and had measured height. We did not use self-reported height because it is subject to systematic bias that varies by geography, time, age, sex, and socioeconomic characteristics like education and ethnicity (Engstrom et al., 2003; Connor Gorber et al., 2007; Wetmore and Mokdad, 2012; Schenker et al., 2010; Ezzati et al., 2006; Clarke et al., 2014; Hayes et al., 2011).

Data sources were included in the NCD-RisC database if:

- measured data on height, weight, waist circumference, or hip circumference were available;
- study participants were five years of age and older;
- data were collected using a probabilistic sampling method with a defined sampling frame;
- data were representative of the general population at the national, subnational, or community level;
- data were from the countries and territories listed in Supplementary file 1.

We excluded data sources on population subgroups whose anthropometric status may differ systematically from the general population, including:

- studies that had included or excluded people based on their health status or cardiovascular risk;
- ethnic minorities;
- specific educational, occupational, or socioeconomic subgroups of the population; and
- those recruited through health facilities, with the exception noted below.

We used school-based data in countries where secondary school enrolment was 70% or higher, and used data whose sampling frame was health insurance schemes in countries where at least 80% of the population were insured. We used data collected through general practice and primary care clinics in high-income countries with universal insurance, because contact with the primary care systems tends to be at least as good as response rates for population-based surveys. No studies were excluded based on the level of height.

We used multiple routes for identifying and accessing data. We accessed publicly available population-based multi-country and national measurement surveys (e.g., Demographic and Health Surveys, and surveys identified via the Inter-University Consortium for Political and Social Research and European Health Interview & Health Examination Surveys Database) as well as the World Health Organization (WHO) STEPwise approach to Surveillance (STEPS) surveys. We requested identification and access to population-based data sources from ministries of health and other national health agencies, via WHO and its regional offices. Requests were also sent via the World Heart Federation to its national partners. We made a similar request to the NCD Risk Factor Collaboration (NCD-RisC; www.ncdrisc.org), a worldwide network of health researchers and practitioners working on NCD risk factors.

To identify major sources not accessed through the above routes, we searched and reviewed published studies. Specifically, we searched Medline (via PubMed) for articles published between 1st January 1950 and 12th March 2013 using the search terms 'body size'[mh:noexp] OR 'body height'[mh:noexp] OR 'body weight'[mh:noexp] OR 'birth weight'[mh:noexp] OR 'overweight'[mh:noexp] OR 'obesity'[mh] OR 'thinness'[mh:noexp] OR 'Waist-Hip Ratio'[mh:noexp] or 'Waist Circumference'[mh:noexp] or 'body mass index' [mh:noexp]) AND ('Humans'[mh]) AND('1950'[PDAT]: '2013'[PDAT]) AND ('Health Surveys'[mh] OR 'Epidemiological Monitoring'[mh] OR 'Prevalence'[mh]) NOT Comment[ptyp] NOT Case Reports[ptyp].

Articles were screened according to the inclusion and exclusion criteria described above. The number of articles identified and retained is summarised in Supplementary file 2. As described above, we contacted the corresponding authors of all eligible studies and invited them to join NCD-RisC. We did similar searches for other cardio-metabolic risk factors including blood pressure, serum cholesterol, and blood glucose. All eligible studies were invited to join NCD-RisC and were requested to analyse data on all cardio-metabolic risk factors.

Anonymised individual record data from sources included in NCD-RisC were re-analysed by the Pooled Analysis and Writing Group or by data holders according to a common protocol. All re-analysed data sources included mean height in standard age groups (18 years, 19 years, 20–29 years, followed by 10 year age groups and 80+ years), as well as sample sizes and standard errors. All analyses incorporated appropriate sample weights and complex survey design when applicable. To ensure summaries were prepared according to the study protocol, the Pooled Analysis and Writing Group provided computer code to NCD-RisC members who requested assistance. We also recorded information about the study population, period of measurement and sampling approach. This information was used to establish that each data source was population-based, and to assess whether it covered the whole country, multiple subnational regions, or one or a small number of communities, and whether it was rural, urban, or combined. All submitted data were checked by at least two independent members of the Pooled Analysis and Writing Group to ensure that their sample selection met the inclusion criteria and that height was measured and not self-reported. Questions and clarifications about sample design and measurement method were discussed with the Collaborating Group members and resolved before data were incorporated in the database. We also extracted data from additional national health surveys, one subnational STEPS survey, and six MONICA sites from published reports.

We identified duplicate data sources by comparing studies from the same country and year. Additionally, NCD-RisC members received the list of all data sources in the database and were asked to ensure that the included data from their country met the inclusion criteria and that there were no duplicates. Data sources used in our analysis are listed in Supplementary file 3.

In this paper, we used data on height in adulthood (18 years of age and older) from the NCD-RisC database for participants born between 1896 and 1996. We used 1472 population-based data sources with measurements on over 18.6 million adults born between 1896 and 1996 whose height had been measured. We did not use data from the 1860–1895 cohorts because data on these early cohorts were available for only six countries (American Samoa, India, Japan, Norway, Switzerland and USA). We had data for 179 of the 200 countries for which estimates were made; these 179 countries covered 97% of the world’s population. All countries had some data on people born after 1946 (second half of analysis period); 134 had data on people born between 1921 and 1945; and 72 had data on people born in 1920 or earlier. Across regions, there were between an average of 2.0 data sources per country in the Caribbean to 34 sources per country in high-income Asia Pacific. 1108 sources had data on men as well as women, 153 only on men, and 211 only on women.

### Statistical methods

The statistical method is described in detail elsewhere (Danaei et al., 2011; Finucane et al., 2014). In summary, the model had a hierarchical structure in which estimates of mean height for each country and year were nested in regional levels and trends, which were in turn nested in those of super-regions and worldwide. In this structure, estimates of mean height for each country and year were informed by its own data, if available, and by data from other years in the same country and in other countries, especially those in the same region with data for similar time periods. The hierarchical structure shares information to a greater degree when data are non-existent or weakly informative (e.g., because they have a small sample size), and to a lesser extent in data-rich countries and regions.

We used birth cohort as the time scale of analysis. We calculated the birth cohort for each observation by subtracting the mid-age of its age group from the year in which data were collected. We modelled trends in height by birth cohort as a combination of linear and non-linear trends, both with a hierarchical structure; the non-linear trend was specified using a second-order random walk (Rue and Held, 2005). The model also included a term that allowed each birth cohort’s height to change as it aged, e.g., because there is gradual loss of height during ageing and because as a cohort ages those who survive may be taller. The model described by Finucane et al (Finucane et al., 2014) had used a cubic spline for age associations of risk factor levels. In practice, the estimated change in population mean height over age was linear with a small slope of over 0.2 cm shorter for men and 0.3 cm shorter for women with each decade of older age. Therefore, we used a linear specification for computational efficiency.

While all our data were from samples of the general population, 796 (54%) of data sources represented national populations, another 199 (14%) major sub-national regions (e.g., one or more provinces or regions of a country), and the remaining 477 (32%) one or a small number of communities. The model accounted for the fact that sub-national and community studies, while informative, might systematically differ from nationally representative ones, and also have larger variation relative to the true values than national studies (e.g., see data from China, India, Japan and the UK in Figure 6 and Figure 7).

We fitted the Bayesian model with the Markov chain Monte Carlo (MCMC) algorithm. We monitored mixing and convergence using trace plots and Brooks–Gelman–Rubin diagnostics (Brooks and Gelman, 1998). We obtained 5000 post burn-in samples from the posterior distribution of model parameters, used to obtain the posterior distribution of mean height. The reported credible intervals represent the 2.5th–97.5th percentiles of the posterior distribution. We report mean height at age 18 years for each birth cohort; heights at other ages are available from the authors. All analyses were done separately by sex because height and its trends over time may differ between men and women.

We tested how well our statistical model predicts missing values by removing data from 10% of countries with data (i.e., created the appearance of countries with no data where we actually had data). The countries whose data were withheld were randomly selected from the following three groups: data-rich (more than 25 cohorts of data, with at least five cohorts after 1960), data-poor (up to and including 12 cohorts of data for women and 8 cohorts for men), and average data availability (13 to 25 cohorts for women, 9 to 25 cohorts for men, or more than 25 cohorts in total with fewer than five after 1960). In total, there were 64 data-rich countries for women and 51 for men; 57 data-poor countries for women and 58 for men; and 56 countries for women and 60 for men that had average data availability. We fitted the model to the data from the remaining 90% of countries and made estimates of the held-out observations. We repeated the test five times, holding out a different subset of data in each repetition. We calculated the differences between the held-out data and the estimates. We also checked the 95% credible intervals of the estimates; in a model with good external predictive validity, 95% of held-out values would be included in the 95% credible intervals.

Our model performed extremely well; specifically, the estimates of mean height were unbiased as evidenced with median errors that were very close to zero globally, and less than ±0.2 cm in every subset of withheld data (Supplementary file 4). Even the 25th and 75th percentiles of errors rarely exceeded ±1 cm. Median absolute error was only about 0.5 cm, and did not exceed 1.0 cm in subsets of withheld data. The 95% credible intervals of estimated mean heights covered 97% of true data for both men and women, which implies good estimation of uncertainty; among subgroups of data, coverage was never < 90%.

## Additional files

Supplementary file 1. Regions used for the Bayesian hierarchical model such that information was shared among countries within each region, among regions in a super-region, and among super-regions in the world. Numbers in brackets show number of countries in each region or super-region. **DOI:** http://dx.doi.org/10.7554/eLife.13410.014

Supplementary file 2. Flowchart of secondary search for data sources. **DOI:** http://dx.doi.org/10.7554/eLife.13410.015

Supplementary file 3. Data sources used in the study, by country. **DOI:** http://dx.doi.org/10.7554/eLife.13410.016

Supplementary file 4. Results of model validation. The validation procedure is described in the main text. **DOI:** http://dx.doi.org/10.7554/eLife.13410.017
