# Supplementary material for: A century of trends in adult human height
Source: eLife. 2016 Jul 26;5:e13410. doi: 10.7554/eLife.13410 (PMC4961475; doi:10.7554/eLife.13410)
Supplement: Supplementary file 1. — Numbers in brackets show number of countries in each region or super-region. DOI: http://dx.doi.org/10.7554/eLife.13410.014 [file elife-13410-supp1.docx]

Libya, Morocco, Occupied Palestinian Territory, Oman, Qatar, Saudi Arabia, Syrian Arab Republic,

Lucia, Saint Vincent and the Grenadines, Suriname, Trinidad and Tobago

Hungary, Macedonia (TFYR), Montenegro, Poland, Romania, Serbia, Slovakia, Slovenia

* Although high-income English-speaking countries are geographically separated, they exhibit remarkably similar trends in

epidemiological risk factors and outcomes. They were therefore grouped together so that the statistical model shares information amongst them more than it does with other countries that are geographically closer but epidemiologically more distinct.

**Super-region**

**Region**

Sub-Saharan Africa (48)

Central Africa (6): Angola, Central African Republic, Congo, DR Congo, Equatorial Guinea, Gabon

East Africa (17): Burundi, Comoros, Djibouti, Eritrea, Ethiopia, Kenya, Madagascar, Malawi, Mauritius, Mozambique, Rwanda, Seychelles, Somalia, Sudan, Tanzania, Uganda, Zambia

Southern Africa (6): Botswana, Lesotho, Namibia, South Africa, Swaziland, Zimbabwe

West Africa (19): Benin, Burkina Faso, Cabo Verde, Cameroon, Chad, Cote d'Ivoire, Gambia,

Ghana, Guinea, Guinea Bissau, Liberia, Mali, Mauritania, Niger, Nigeria, Sao Tome and Principe, Senegal, Sierra Leone, Togo

Central Asia, Middle East and

North Africa (28)

Central Asia (9): Armenia, Azerbaijan, Georgia, Kazakhstan, Kyrgyzstan, Mongolia, Tajikistan, Turkmenistan, Uzbekistan

Middle East and North Africa (19): Algeria, Bahrain, Egypt, Iran, Iraq, Jordan, Kuwait, Lebanon,

Tunisia, Turkey, United Arab Emirates, Yemen

South Asia (6)

South Asia (6): Afghanistan, Bangladesh, Bhutan, India, Nepal, Pakistan

East and South East Asia (16)

East Asia (4): China, China (Hong Kong SAR), North Korea, Taiwan

South East Asia (12): Brunei Darussalam, Cambodia, Indonesia, Lao PDR, Malaysia, Maldives, Myanmar, Philippines, Sri Lanka, Thailand, Timor-Leste, Viet Nam

Oceania (17)

Oceania (17): American Samoa, Cook Islands, Fiji, French Polynesia, Kiribati, Marshall Islands,

Micronesia (Federated States of), Nauru, Niue, Palau, Papua New Guinea, Samoa, Solomon Islands, Tokelau, Tonga, Tuvalu, Vanuatu

High-income Asia Pacific (3)

High-income Asia Pacific (3): Japan, Singapore, South Korea

Latin America and Caribbean

(35)

Andean Latin America (3): Bolivia, Ecuador, Peru

Caribbean (18): Antigua and Barbuda, Bahamas, Barbados, Belize, Bermuda, Cuba, Dominica,

Dominican Republic, Grenada, Guyana, Haiti, Jamaica, Puerto Rico, Saint Kitts and Nevis, Saint

Central Latin America (9): Colombia, Costa Rica, El Salvador, Guatemala, Honduras, Mexico, Nicaragua, Panama, Venezuela

Southern Latin America (5): Argentina, Brazil, Chile, Paraguay, Uruguay

High-income Western countries (27)

High-income English-speaking countries* (6): Australia, Canada, Ireland, New Zealand, United

Kingdom, United States of America

North Western Europe (12): Austria, Belgium, Denmark, Finland, Germany, Greenland, Iceland, Luxembourg, Netherlands, Norway, Sweden, Switzerland

South Western Europe (9): Andorra, Cyprus, France, Greece, Israel, Italy, Malta, Portugal, Spain

Central and Eastern Europe

(20)

Central Europe (13): Albania, Bosnia and Herzegovina, Bulgaria, Croatia, Czech Republic,

Eastern Europe (7): Belarus, Estonia, Latvia, Lithuania, Moldova, Russian Federation, Ukraine
