# Supplementary material for: A century of trends in adult human height
Source: eLife. 2016 Jul 26;5:e13410. doi: 10.7554/eLife.13410 (PMC4961475; doi:10.7554/eLife.13410)
Supplement: Supplementary file 2. — DOI: http://dx.doi.org/10.7554/eLife.13410.015 [file elife-13410-supp2.docx]

24,202 citations retrieved for 1950‐2013

(Medline)

16,738 excluded after title and abstract review, because they did not contain relevant data, or because data were from non‐random and/or non‐ representative samples

7,464 kept for full‐text

review

1,584 could not retrieve full‐text

1,363 because they did not contain relevant data, or because data were from non‐random and/or non‐

representative samples

3,196 remaining after full‐

text review

700 because contained only self‐

reported data

141 because they were in languages other than English, Spanish, Portuguese, Chinese, Italian, French, and Farsi which were accessible to reviewers

260 because the sample consisted of children under five years of age

220 because data were already accessed via primary data access
