# Supplementary material for: A century of trends in adult human height
Source: eLife. 2016 Jul 26;5:e13410. doi: 10.7554/eLife.13410 (PMC4961475; doi:10.7554/eLife.13410)
Supplement: Supplementary file 4. — The validation procedure is described in the main text. DOI: http://dx.doi.org/10.7554/eLife.13410.017 [file elife-13410-supp4.docx]

**Men**

| **Data** | | **No. of held out observations** | **Percent covered** | **Error (cm)^†^** | | | | **Absolute error** | | | |
| --- | --- | --- | --- | --- | --- | --- | --- | --- | --- | --- | --- |
|  |  |  |  | **Median** | **Q1** | **Q3** | **(p*)** | **Median** | **Q1** | **Q3** | **(p*)** |
| All | | 2986 | 97 | 0.011 | -0.494 | 0.554 | 0.73 | 0.53 | 0.23 | 1.02 | 0.73 |
| Super-region | Central and Eastern Europe | 214 | 100 | 0.045 | -0.422 | 0.394 | 0.98 | 0.42 | 0.17 | 0.84 | 0.98 |
|  | Central Asia, Middle East and North Africa | 279 | 96 | -0.031 | -0.468 | 0.676 | 0.74 | 0.58 | 0.26 | 1.12 | 0.74 |
|  | East and South East Asia | 150 | 95 | -0.015 | -0.505 | 0.451 | 0.87 | 0.50 | 0.19 | 0.83 | 0.87 |
|  | High-income Asia Pacific | 585 | 95 | 0.021 | -0.529 | 0.785 | 0.69 | 0.63 | 0.26 | 1.20 | 0.69 |
|  | High-income Western countries | 1157 | 98 | -0.011 | -0.461 | 0.484 | 0.85 | 0.47 | 0.21 | 0.90 | 0.85 |
|  | Latin America and Caribbean | 242 | 96 | 0.055 | -0.581 | 0.576 | 0.89 | 0.58 | 0.26 | 1.08 | 0.89 |
|  | Oceania | 147 | 95 | -0.030 | -0.779 | 0.760 | 0.94 | 0.77 | 0.39 | 1.42 | 0.94 |
|  | South Asia | 32 | 97 | 0.025 | -0.648 | 0.289 | 0.69 | 0.49 | 0.12 | 1.00 | 0.69 |
|  | Sub-Saharan Africa | 180 | 98 | 0.049 | -0.441 | 0.449 | 0.90 | 0.44 | 0.21 | 0.94 | 0.90 |
| Urban or rural studies | Rural | 359 | 94 | 0.008 | -0.661 | 0.730 | 0.99 | 0.70 | 0.33 | 1.58 | 0.99 |
|  | Urban | 704 | 98 | 0.016 | -0.541 | 0.527 | 1.00 | 0.53 | 0.24 | 1.02 | 1.00 |
|  | Both rural and urban | 1923 | 97 | 0.010 | -0.449 | 0.540 | 0.65 | 0.49 | 0.21 | 0.95 | 0.65 |
| Study representativeness | Community | 952 | 97 | 0.025 | -0.595 | 0.585 | 0.99 | 0.59 | 0.28 | 1.16 | 0.99 |
|  | Sub-national | 389 | 97 | 0.021 | -0.436 | 0.476 | 0.94 | 0.46 | 0.20 | 0.91 | 0.94 |
|  | National | 1645 | 97 | -0.003 | -0.467 | 0.542 | 0.66 | 0.50 | 0.22 | 0.96 | 0.66 |
| Age band | 18-40 | 1197 | 96 | 0.085 | -0.474 | 0.724 | 0.38 | 0.58 | 0.26 | 1.11 | 0.38 |
|  | 40-60 | 909 | 98 | -0.152 | -0.599 | 0.248 | 0.49 | 0.42 | 0.20 | 0.80 | 0.49 |
|  | 60 and above | 880 | 96 | 0.093 | -0.415 | 0.668 | 0.61 | 0.55 | 0.25 | 1.11 | 0.61 |
| Years | 1896-1921 | 237 | 94 | 0.164 | -0.489 | 1.063 | 0.40 | 0.72 | 0.33 | 1.60 | 0.40 |
|  | 1922-1946 | 999 | 97 | -0.021 | -0.468 | 0.426 | 0.97 | 0.45 | 0.21 | 0.89 | 0.97 |
|  | 1947-1971 | 1086 | 98 | -0.003 | -0.482 | 0.525 | 0.83 | 0.51 | 0.22 | 0.90 | 0.83 |
|  | 1972-1996 | 664 | 95 | 0.059 | -0.551 | 0.766 | 0.53 | 0.67 | 0.27 | 1.25 | 0.53 |
| Data density | Data poor | 172 | 99 | 0.010 | -0.518 | 0.479 | 0.97 | 0.52 | 0.23 | 0.95 | 0.97 |
|  | Average data density | 490 | 97 | 0.049 | -0.442 | 0.552 | 0.86 | 0.49 | 0.20 | 1.02 | 0.86 |
|  | Data rich | 2324 | 97 | 0.000 | -0.504 | 0.556 | 0.69 | 0.53 | 0.24 | 1.02 | 0.69 |

**Women**

| **Data** | | **No. of held out observations** | **Percent covered** | **Error (cm)^†^** | | | | **Absolute error** | | | | |
| --- | --- | --- | --- | --- | --- | --- | --- | --- | --- | --- | --- | --- |
|  |  |  |  | **Median** | **Q1** | **Q3** | **(p*)** | **Median** | **Q1** | **Q3** | **(p*)** | |
| All | | 3241 | 97 | -0.003 | -0.450 | 0.495 | 0.76 | 0.47 | 0.21 | 0.84 | 0.76 | |
| Super-region | Central and Eastern Europe | 338 | 99 | 0.015 | -0.429 | 0.445 | 0.97 | 0.44 | 0.21 | 0.80 | 0.97 | |
|  | Central Asia, Middle East and North Africa | 239 | 98 | -0.037 | -0.445 | 0.403 | 0.99 | 0.43 | 0.19 | 0.78 | 0.99 | |
|  | East and South East Asia | 134 | 95 | 0.056 | -0.447 | 0.606 | 0.96 | 0.57 | 0.28 | 1.00 | 0.96 | |
|  | High-income Asia Pacific | 94 | 99 | 0.088 | -0.434 | 0.636 | 0.77 | 0.54 | 0.25 | 0.91 | | 0.77 |
|  | High-income Western countries | 1518 | 97 | -0.036 | -0.496 | 0.507 | 0.69 | 0.50 | 0.23 | 0.88 | 0.69 | |
|  | Latin America and Caribbean | 342 | 98 | 0.030 | -0.414 | 0.458 | 0.94 | 0.43 | 0.19 | 0.79 | 0.94 | |
|  | Oceania | 155 | 96 | 0.088 | -0.497 | 0.697 | 0.81 | 0.59 | 0.28 | 1.23 | 0.81 | |
|  | South Asia | 13 | 100 | -0.011 | -0.514 | 0.310 | 0.80 | 0.45 | 0.28 | 0.58 | 0.80 | |
|  | Sub-Saharan Africa | 408 | 99 | 0.016 | -0.295 | 0.389 | 0.66 | 0.34 | 0.15 | 0.66 | 0.66 | |
| Urban or rural studies | Rural | 216 | 94 | 0.035 | -0.593 | 0.762 | 0.83 | 0.67 | 0.29 | 1.55 | 0.83 | |
|  | Urban | 799 | 99 | 0.006 | -0.469 | 0.480 | 0.99 | 0.47 | 0.23 | 0.85 | 0.99 | |
|  | Both rural and urban | 2226 | 97 | -0.006 | -0.432 | 0.472 | 0.75 | 0.45 | 0.20 | 0.80 | 0.75 | |
| Study representativeness | Community | 863 | 97 | 0.022 | -0.514 | 0.515 | 0.90 | 0.52 | 0.24 | 0.95 | 0.90 | |
|  | Sub-national | 432 | 98 | 0.013 | -0.487 | 0.524 | 0.85 | 0.51 | 0.24 | 0.89 | 0.85 | |
|  | National | 1946 | 97 | -0.013 | -0.421 | 0.468 | 0.82 | 0.44 | 0.19 | 0.79 | 0.82 | |
| Age band | 18-40 | 1443 | 98 | 0.068 | -0.349 | 0.594 | 0.22 | 0.48 | 0.21 | 0.87 | 0.22 | |
|  | 40-60 | 964 | 99 | -0.142 | -0.523 | 0.206 | 0.19 | 0.37 | 0.18 | 0.64 | 0.19 | |
|  | 60 and above | 834 | 94 | 0.068 | -0.520 | 0.724 | 0.63 | 0.62 | 0.28 | 1.07 | 0.63 | |
| Years | 1896-1921 | 174 | 90 | -0.085 | -0.727 | 1.119 | 0.74 | 0.98 | 0.53 | 1.55 | 0.74 | |
|  | 1922-1946 | 1006 | 97 | -0.060 | -0.523 | 0.445 | 0.72 | 0.49 | 0.23 | 0.84 | 0.72 | |
|  | 1947-1971 | 1186 | 99 | 0.013 | -0.368 | 0.450 | 0.67 | 0.42 | 0.17 | 0.71 | 0.67 | |
|  | 1972-1996 | 875 | 98 | 0.020 | -0.435 | 0.523 | 0.63 | 0.48 | 0.22 | 0.90 | 0.63 | |
| Data density | Data poor | 237 | 100 | 0.025 | -0.342 | 0.514 | 0.88 | 0.41 | 0.18 | 0.75 | 0.88 | |
|  | Average data density | 591 | 97 | 0.007 | -0.375 | 0.405 | 0.86 | 0.39 | 0.16 | 0.81 | 0.86 | |
|  | Data rich | 2413 | 97 | -0.009 | -0.485 | 0.509 | 0.74 | 0.50 | 0.23 | 0.86 | 0.74 | |
